# The state of the art in telerehabilitation for musculoskeletal conditions

**DOI:** 10.1186/s40945-022-00155-0

**Published:** 2023-01-04

**Authors:** Marina P. Baroni, Maria Fernanda A. Jacob, Wesley R. Rios, Junior V. Fandim, Lívia G. Fernandes, Pedro I. Chaves, Iuri Fioratti, Bruno T. Saragiotto

**Affiliations:** 1 Masters and Doctoral Program in Physical Therapy, Universidade Cidade de São Paulo, São Paulo, São Paulo 03071-000 Brazil; 2 Centre for Pain, Health and Lifestyle, São Paulo São Paulo, Brazil; 3 Discipline of Physiotherapy, Graduate School of Health, University of Technology Sydney, NSW 2000 Sydney, Australia

**Keywords:** Telehealth, Chronic pain, Physiotherapy

## Abstract

**Background:**

Given the rapid advances in communication technology and the need that emerged from the COVID-19 pandemic, telehealth initiatives have been widely used worldwide. This masterclass aims to provide an overview of telerehabilitation for musculoskeletal conditions, synthesizing the different terminologies used to describe telehealth and telerehabilitation, its effectiveness and how to use it in clinical practice, barriers and facilitators for the implementation in health services, and discuss the need of a curriculum education for the near future.

**Main body:**

Telerehabilitation refers to the use of information and communication technologies provided by any healthcare professionals for rehabilitation services. Telerehabilitation is a safe and effective option in the management of musculoskeletal conditions in different models of delivery. There are many technologies, with different costs and benefits, synchronous and asynchronous, that can be used for telerehabilitation: telephone, email, mobile health, messaging, web-based systems and videoconferences applications. To ensure a better practice of telerehabilitation, the clinician should certify safety and access, and appropriateness of environment, communication, technology, assessment, and therapeutic prescription. Despite the positive effect of telerehabilitation in musculoskeletal disorders, a suboptimal telerehabilitation implementation may have happened due to the COVID-19 pandemic, especially in countries where telehealth was not a reality, and clinicians lacked training and guidance. This emphasizes the need to identify the necessary curriculum content to guide future clinicians in their skills and knowledge for telerehabilitation. There are some challenges and barriers that must be carefully accounted for to contribute to a health service that is inclusive and relevant to health professionals and end users.

**Conclusions:**

Telerehabilitation can promote patient engagement in health care and plays an important role in improving health outcomes in patients with musculoskeletal conditions. Digital health technologies can also offer new opportunities to educate patients and facilitate the process of behavior change to a healthy lifestyle. Currently, the main needs in telerehabilitation are the inclusion of it in health curriculums in higher education and the development of cost-effectiveness and implementation trials, especially in low- and middle-income countries where access, investments and digital health literacy are limited.

## Background

The first mention of terminologies related to technology and healthcare in scientific publications was in the 1970s, using the terms telemedicine and telehealth. Later in the 1990s, terms such as e-health and telerehabilitation emerged with the advance in technology options and modalities [1, 2]. A bibliometric analysis of the trends in the use of telehealth terms from 2012 concluded that the most cited terms (in titles and abstracts) are telemedicine (8,028 citations), e-health (2,573 citations), and telehealth (1,679 citations) and telerehabilitation (~ 350 citations) [1].

In the literature, the terms telehealth, telemedicine, e-health and telerehabilitation are used interchangeably as similar or equivalent [1, 3, 4]. However, recent studies have highlighted the large diversity of telehealth terms over time [5, 6]. In 2021, a scoping review synthesized the existing telehealth evidence in geriatric care during the COVID-19 pandemic [6] and the authors identified 49 different terminologies related to telehealth present in 79 studies. Previously in 2020, a scoping review synthesized the existing telehealth evidence from the COVID-19 pandemic onset [5]. This scoping review included 543 studies, identifying 39 different terminologies related to telehealth services in 42 countries. The most common terms and definitions were telehealth, telemedicine, telerehabilitation, and digital physical therapy. In addition, the authors also observed some heterogeneity in the definition of these terms, leading to different meanings. A global consensus on terms and definitions in telehealth is needed and has high impact on telehealth research agenda.

Telehealth is considered an umbrella term for all healthcare services [3, 7–9], defined by the World Health Organization as “the delivery of health care services, where distance is a critical factor, by all health care professionals using information and communication technologies for the exchange of valid information for the diagnosis, treatment and prevention of disease and injuries, research and evaluation, and for the continuing education of health care providers, all in the interests of advancing the health of individuals and their communities” [10, 11]. Telemedicine restricts telehealth use to physicians [12] and, telerehabilitation refers to the services provided by any healthcare professionals for rehabilitation services [8]. Telerehabilitation is a subfield of telehealth and is considered an emerging concept in rehabilitation services that have been attracting attention worldwide [2, 9]. Telerehabilitation's first publication was in the 1990s [2], and the term was defined as: “the delivery of rehabilitation services via information and communication technologies” [8]. Table 1 presents a synthesis of the terminologies and definitions most used in telehealth literature. For this masterclass, we will use the term telerehabilitation.

**Table 1:** Most common telehealth-related terminologies followed by each definition

Terminology	Definition
Digital health	“Digital health is defined as the use of digital, mobile and wireless technologies to support the achievement of health objectives. Digital health describes the general use of information and communications technologies (ICT) for health and is inclusive of both mHealth and eHealth” [5]
Digital physical therapy practice	“Digital physical therapy practice is defined as the health care services, support, and information provided remotely via digital communication and devices” [5]
Digital therapeutics (DTx)	“Digital therapeutics (DTx) deliver evidence-based therapeutic interventions to patients that are driven by high quality software programs to prevent, manage, or treat a medical disorder or disease. They are used independently or together with medications, devices, or other therapies to optimize patient care and health outcomes. DTx products incorporate advanced technology best practices relating to design, clinical validation, usability, and data security” [13]
Electronic health (eHealth)	“The cost-effective and secure use of information and communications technologies in support of health and health-related fields, including health-care services, health surveillance, health literature, and health education, knowledge and research” [13, 14]
Mobile health (mHealth)	“mHealth or mobile as medical and public health practice supported by mobile devices, such as mobile phones, patient monitoring devices, personal digital assistants (PDAs), and other wireless devices” [13, 15]
Telecare	“The means by which technologies and related services at a distance are accessed by or provided for people and/or their carers at home or in the wider community, in order to facilitate empowerment or the provision of care and/or support in relation to needs associated with their health and well-being” [16]
Teleconsultation	“Teleconsultation refers to the electronic communication between a physician and a patient or between two physicians for the purpose of diagnosis and/or treatment” [5]
Telehealth	“Telehealth is defined as the delivery of health care services, where patients and providers are separated by distance. Telehealth uses ICT for the exchange of information for the diagnosis and treatment of diseases and injuries, research, and evaluation, and for the continuing education of health professionals. Telehealth can contribute to achieving universal health coverage by improving access for patients to quality, cost-effective, health services wherever they may be. It is particularly valuable for those in remote areas, vulnerable groups and ageing populations” [11]
Telemedicine	“Telemedicine is defined as the delivery of health care services, where distance is a critical factor, by all health care professionals using information and communication technologies for the exchange of valid information for diagnosis, treatment and prevention of disease and injuries, research and evaluation, and for the continuing education of health care providers, all in the interests of advancing the health of individuals and their communities” [3]
Telerehabilitation	“Telerehabilitation refers to the delivery of rehabilitation services via information and communication technologies” [8]
Remote Monitoring	“The use of devices that collect patient/user vital sign and other data and effect its transmission, in real-time or periodically, to a monitoring centre” [16]
Tele-assistance	“The assistance given when a health professional or other person, at the location of the user/patient (the originating site), assists the carrying out of a medical act guided by a doctor or other health professional at the service provider site” [16]
Tele-intervention	“Tele-intervention is a therapeutic medical act which is performed remotely by a physician on a patient, without or with the local presence of other healthcare professional (e.g. telesurgery)” [16]
Telemonitoring	“The use of communications technologies to remotely collect/send data relevant to the health and well-being of a user / patient to a monitoring centre to assist in diagnosis and monitoring” [16]
Telephysiotherapy	“Telephysiotherapy is a provision of physiotherapy services at a distance, using telecommunication technology such as video conferencing or telephone meeting, when an in-person visit is not a feasible option “ [17]
Remote treatment or therapy	“Meeting with a patient through telephone, cellular phone, the internet, or other electronic media in place of or in addition to conventional face-to-face visits to deliver treatment (term is most often used in psychotherapy)” [13]
Telephone intervention	“Telephone intervention is defined as an intervention that enables healthcare professionals to verbally communicate remotely with caregivers. A healthcare professional is a trained healthcare person who has received specific healthcare education and training in the management and care of people with diagnosed conditions, their family members, significant others or caregivers (e.g., nurses, medical doctors, social workers, physiotherapists, occupational therapists, counsellors/psychologists, and dieticians/nutritionists)” [18]
Telementoring	“The use of audio, video, and other telecommunications technologies to provide guidance or direction” [16]
Virtual care	“Virtual care is defined as any interaction occurring remotely between patients and/or members of their circle of care, through any form of communication or information technology with the aim of facilitating or maximizing the quality and effectiveness of patient care” [5]
Videoconferencing	“Real-time two way transmission of digitised video images between two or more locations”
Virtual support	“Clinical and community supports involving broadly increased in-home acute and primary care” [5]

## The effectiveness of telerehabilitation in musculoskeletal conditions

Given the rapid advances in communication technology and the need that emerged from the COVID-19 pandemic, telerehabilitation has become increasingly popular and has been tested for many health conditions, such as neurological [19, 20], cardiopulmonary [21–24], musculoskeletal [25–30], post-operatory conditions [31] metabolic disorders (obesity and/or diabetes) [32–34] and cancer [34–36]. Telerehabilitation allows the health care service to be offered through a range of modalities of delivery, that corresponds to the moment that information is transmitted. Synchronous modality is the term used to describe telerehabilitation that happens simultaneously with regard to the transmission of data, speech and information, also known as real-time (e.g., video-conference) [4, 16]. Asynchronous modality is the term used to describe a type of telerehabilitation consultation that does not occur simultaneously. The health professional use digital images stored and forwarded to assist in diagnosis or treatment [16]. Finally, a hybrid model consists of alternating in-person and synchronous or asynchronous telerehabilitation [37]. Table 2 presents the current evidence based on systematic reviews from different modalities of telerehabilitation.

**Table 2:** Summary of effectiveness in different modalities of telerehabilitation in musculoskeletal disorders

Author, year	Population	Physical therapy intervention	Modality of delivery	Outcomes		Results
Cottrell et al., 2017 [26]	Any diagnosed primary musculoskeletal condition	Real-time telerehabilitation *vs.* face-to-face treatment or usual care	Synchronous: real-time telerehabilitation (telephone, videoconferencing software)	Pain, quality of life, disability or function (physical, social or psychological)	Real-time telerehabilitation appears to be superior for the improvement of physical function and reducing disability; and similar in reducing pain and psychological function	
Wang et al., 2019 [28]	Total hip or knee replacement	Tecnology-based interventions *vs.* usual care or no intervention	Mixed (synchronous and asynchronous): telerehabilitation (phone, videoconferencing; game-based therapy; web-based therapy; or virtual reality devices	Pain and function	Technology-based interventions is superior in reducing pain and improving function	
Du et al., (2020) [38]	Chronic low back pain	e-Health based self-management programs *vs.* waiting-list, usual care, or face-to-face health education	Asynchronous: internet-based and mobile-health	Pain and function	e-Health based self-management was superior in reducing pain intensity and improving function in short-term period	
Lima et al., 2021 [30]	Adults with musculoskeletal pain	Web-based pain education *vs*. minimal intervention (no intervention or booklet) or usual care	Assynchronous: web-based education	Pain and function	Web-based pain education reduce pain and disability compared with minimal intervention	
Chen et al., (2021) [39]	Low back pain	m-Health and usual care *vs.* usual care alone	Mixed (synchronous or asynchronous): Telephone calls; text message; mobile phone software, motion sensor biofeedback, and network-based game consoles	Pain and function	The use of simultaneous m-Health and usual care interventions is superior in reducing pain intensity and improving function Subgroup analyses showed that mHealth using telephone calls is better than mobile health without the use of telephone calls	
Lara-Palomo et al., (2022) [40]		e-Health intervention based on self-maintenance and education *vs.* face-to-face intervention (minimal intervention, physical therapy), and nonintervention control groups	Mixed (synchronous and asynchronous): e-Health (information, computer, and communication technology)	Pain, function and quality of life	e-Health interventions based on self-maintenance and education are as effective on pain and function status as face-to-face or home-based interventions	

Previous systematic reviews have shown that telerehabilitation is superior to usual care, minimal intervention or waiting list controls [28, 30, 38, 39], and similar to face-to-face interventions in reducing pain and improving function in patients with musculoskeletal disorders [26, 40]. Telerehabilitation also seems to provide similar outcomes to face-to-face intervention and usual care for improving quality of life [26, 40] and psychological function [26]. Apparently, the type of intervention (e.g., education, exercise, physical therapy, or self-management), the mode of delivery (synchronous or asynchronous), or the telecommunication technology (e.g., telephone, text message, videoconferencing, applications, website) does not influence the estimates and previous studies did not include enough trials to conduct subgroup analyses. To date, clinicians should consider the telerehabilitation modality and technology that can be best applied to the patient’s preference considering access and digital literacy. They should keep choosing evidence-based interventions for chronic pain that can be applied through telerehabilitation, such as exercise and education [41–44].

Even though the numerous options of interventions and modalities in telerehabilitation, it is necessary to prioritize patient-centered care, considering the preference of the user of health service. In this context, some studies have shown excellent patient satisfaction, with no difference when compared to presential care [45, 46]. These results emphasize that the humanization of care is possible even in the telerehabilitation modality, since it refers to the approach of the patient by the health professional and access to health, and not to the modality itself [45, 46]. In this way, patient satisfaction is higher when the telerehabilitation modality offers simultaneous contact with the physiotherapist [47–49].

Despite the positive effect of telerehabilitation in musculoskeletal disorders, studies that evaluate the cost-effectiveness of digital solutions in musculoskeletal conditions are scarce. It is necessary to investigate the cost-effectiveness of this modality and hybrid models, especially in low- and middle-income countries, considering that different technologies add different values to the intervention [50]. A study by Cuperus et al. (2016) [51] showed better economic evaluation from a societal perspective in the face-to-face intervention compared to a telephone-based nonpharmacologic multidisciplinary treatment program for patients with generalized osteoarthritis. Nevertheless, a study by Salisbury et al. (2013) [52] showed that providing physiotherapy by telephone assessment and advice services for patients with musculoskeletal disorders was equally cost-effectiveness compared with usual wait list-based care. Besides that, telephone assessment and advice services for physiotherapy reduce usual waiting list-based care and provide faster access to treatment [52]. The digital health solutions also demonstrate to be economical in delivered of an internet-based cognitive-behavioral intervention for chronic pain [53] and remote orthopedic consultations [54]. Knowing the relationship of cost-effectiveness is important for clinical decision-making and for health managers to implement telerehabilitation services in the health system. Thus, adherence to telerehabilitation requires a significant change in service management and redesign of traditional patient care models. In this context, telerehabilitation can provide increase users’ access to health specialties that are not provided in person in their cities [55].

## The clinical use of telerehabilitation in musculoskeletal conditions

Before starting the telerehabilitation process, the clinician must be aware of whether the patient fits into a group that can perform care online exclusively [56] or in a hybrid model [57]. Complex patients may be managed more successfully if in-person care is performed initially and then telerehabilitation management is provided afterwards (hybrid model) [57]. A few practical points are summarized below to ensure a better adaptation to the process of implementing telehealth in clinical practice.

### Safety

Procedures that address patient safety during an appointment, such as being prepared for medical emergencies, are essential. Identifying the patient's location, family contacts and medical services close to the patient are important procedures to be carried out, especially in cases of elderly or fragile patients [57]. If a patient is at any risk of adverse events during care, it may be important to have a second person be physically present with the patient during sessions. Remote Patient Monitoring with devices technology has been advancing and will become popular in the very near future. Remote Patient Monitoring can allow patients to share their vital signs instantaneously with clinicians (using apps and medical devices) during a session [58]. This may ensure more quality and safety for patient encounters.

### Privacy and digital security

Privacy and digital security are two important issues when it comes to technology, especially when dealing with one’s health. The primary security risk is unauthorized access, by hackers or business companies, to the patients’ data during collection, transmission, or storage [59]. Currently, the Health Insurance Portability and Accounting Act (HIPAA) contains the primary set of regulations that guide the privacy and security of health information [60]. Besides HIPPA, each country has its own data protection laws, and it is important to get familiarized with them. Typically, clinicians are required to obtain patients' informed consent, as well as to use secure and encrypted online service platforms [61]. The informed consent should present legal policies, confidentiality issues and potential risk situations, advantages and possible disadvantages of telerehabilitation related to the use of software technologies [62].

### Access and environment

Before starting an online consultation with the patient, it is important to ensure that patients have access to a proper device (computer or smartphone), stable and good quality broadband internet and familiarity with technology [63]. Clinicians must be aware of patients’ sociodemographic information and previous experiences with technology to best design a telerehabilitation intervention that supports patient participation and engagement [64]. In addition, physical factors such as good local acoustics and space organization where the care will be performed are essential to facilitate care and improve the patient's experience.

### Communication

The most powerful tool of clinicians during telerehabilitation is communication. Establishing a strong therapeutic alliance by building trust and empathy can be challenging in telerehabilitation encounters as sessions tend to be quicker and in a less inviting environment (compared to a clinic). However, clear communication often leads to better engagement and may even result in better outcomes for patients at the end of treatment [56, 65]. Simple strategies, such as keeping eye contact with the patient by looking at the computer’s camera and avoiding performing other tasks while the patient is talking can be important for establishing a connection [66]. Also, there may be audio delays during a conversation with a patient, so it is recommended to wait a few seconds after the patient stops speaking before the clinician begin to speak [67]. Using simple words, simple commands for exercises, and avoiding technical language are also key valuable strategies to use during telerehabilitation [66].

### Assessment

Clinicians should consider grouping certain components of the examination process to minimize the number of times a patient has to change position or camera angles [68]. The clinician can screen share images or videos, with high resolution, with the patient during the assessment to facilitate the self-perform or ask the patient to record themselves [67]. Ideally, the presence of a family member or friend can be useful during the assessment to adjust the camera to accommodate different patient positions (standing, lying, full-body view, etc.) [67, 68]. The personal examination might use a different order or only use certain components, based on the patient’s presenting symptoms [67]. The collection of personal data, history of current illness, previous history of the disease, physical activity level, routine, occupation, signs and symptoms can help guide the assessment. The fact that the patient is often at home during the remote assessment makes it easier for them to demonstrate their pain and functional complaints during their daily activities in their own environment, something that is limited during evaluation in a clinical environment [69].

For the objective physiotherapy assessment, telerehabilitation is feasible with good to excellent concurrent validity and reliability (ICC > 0.90) for most components of physical assessment (i.e., observation, range of motion, muscle strength, gait analysis, special orthopedic and neurodynamic tests) [70]. Unfortunately, no studies had employed the standard error measurement (SEM) and coefficient of variation (CV) in analyzing the absolute reliability of virtual physiotherapy assessments [70]. Therefore, future studies should consider appropriate statistical methods when reporting validity and reliability for components of virtual assessment. The assessment of the range of motion can be measured by asking the patient to align the camera perpendicularly to the performed movement by direct observation, using a universal goniometer on the digital image or through applications that enable the measurement of range of motion in videos [70, 71]. The quantitative voluntary muscle testing can be assessed by using manual resistance by a trained caregiver, a family member, an allied health assistant or the use of the patient’s own resistance [70]. In addition, functional movements should be assessed and recorded, such as sitting and standing, gait, picking up an object from above or bending down to pick up objects [68].

Patients should be asked to demonstrate on video the body region affected, point the area to the pain location and delineate any radiating pain [68]. If necessary, the clinician can request the patient to palpate the region and report the increase of pain or change in sensitivity. To guide the patient to the self-palpation, the clinician can provide a body chart via the telerehabilitation system (or communication platform) ahead of time [70]. In addition, the use of specific orthopedic and neurodynamic tests in an online environment might be improved by guiding and training the patients or caregiver through real-time feedback, supplemented by high-quality video or a video weblink [70]. However, it is important to consider that most specific tests are based on validated physical examination maneuvers performed during face-to-face patient encounters and had been modified to enable the patient to self-perform the maneuvers [68]. Then, this use must be carried out with due care. Before performing the tests, the clinician must consider if the chosen tests possess a good quality of clinimetric properties and are validated for an online environment, if possible, where a patient will perform the test alone [68, 70]. Self-palpation, specific orthopedics and neurodynamic tests can be difficult to be self-executed by the patients due to the self-performed nature of these assessments [70]. The complexity of the tests [70], low camera resolution [72], bad lighting [72], inexperienced assessors [73], lack of video conference [74] and poor rapport [73, 74] could be other factors associated with poor concurrent validity in musculoskeletal conditions.

Although many aspects of a patient’s condition and symptomatology can be assessed through a virtual exam [75, 76], most of them are consensus-based multispecialty guidelines, which have been screened by a committee of national experts for incorporation into the virtual assessment, and are not validated for telehealth use [76]. In more disabled patients, the virtual assessment and tasks requiring independent activities can be more difficult [77]. Some patients with chronic pain conditions may have limited mobility that may require the assistance of the examining provider in performing many functions [77]. These patients can have difficulty exposing a post-procedural incision, performing basic motor tasks, and positioning the camera in a way that provides the most information to the clinician [77]. When the virtual assessment seems to be suboptimal, the clinician and patient should consider a face-to-face visit for the hands-on assessment.

Questionnaires are important tools for the process of evaluating and monitoring patients with musculoskeletal conditions. Technologies used in the online environment facilitate the use of the questionnaires, promoting data storage and data sharing [78]. The clinician can send to the patient the questionnaire to be answered during or after a session and save the patient’s time [79], and store the results online for a proper follow-up [78]. Finally, the clinician must be aware of the signs of serious underlying diseases as the red flags, to gauge the level of disability for pain, develop differential diagnoses and provide counsel to the patient [68].

### Exercise prescription and online education

According to different guidelines, best practice for musculoskeletal rehabilitation includes education and exercises [42–44, 80, 81], both possible to be implemented through telerehabilitation technologies. Patient education can be delivered in standard formats (articles, written messages) or a wide array of multimedia (audio, video, interactive games, videoconference, etc.). This education interaction can promote patient engagement in health care and plays an important role in improving health outcomes and developing self-efficacy [82]. Digital health technologies can also offer new opportunities to educate patients and facilitate the process of behavior change to a healthy lifestyle and monitoring [83].

Delivering exercise therapy via telerehabilitation platforms needs a few adjustments. Clinicians and patients should be allocated in a comfortable, spacious and quiet environment so they can see and hear each other. The clinician must be aware of available materials, demonstrate the exercises or movements that the patient may do, and have time to explain the patient’s doubts about the exercises [66]. A good option for exercising in a home environment is to consider exercises that can use the body's own resistance or that require simple materials (e.g., elastic bands), as well as the ones that can be performed with the use of objects at home (e.g., chair, window, broom). The exercises’ complexity must be considered too, and functional exercises should be prioritized given that they are easy to understand and perform. Any resource that facilitates patient's exercise understanding should be used, such as exercise demonstration and explanation during care, or the use of written material, photos/images, videos, etc. The patient's exercise demonstration to the therapist is an essential part of telerehabilitation so that observations and possible corrections can be made. Characteristics of exercises’ doses and frequencies must always be discussed and established, considering evolutions of loads according to each phase, clinical objectives and musculoskeletal condition [84]. Motivation is also important in telerehabilitation, and the clinician should have strategies for improving engagement and motivation, such as reminders [85], weekly challenges and periodic feedback [86].

## Barriers and challenges of telerehabilitation implementation

Despite promising evidence demonstrating the effectiveness of telerehabilitation initiatives, current uptake within health systems and health policies is suboptimal. Challenges and barriers to telerehabilitation are complex, multilevel, multifactorial, and context-dependent.

One common barrier to telerehabilitation reported by clinicians is the use of hands-on approaches, such as manual therapy, acupuncture, and others [87, 88]. There is evidence that manual therapy can help in the treatment of musculoskeletal pain conditions, and should be used as an adjunct to other evidence-based treatments [56]. Also, hands-on techniques still represent an element of musculoskeletal physiotherapy practice that is well appreciated by patients [89] and clinical professionals [87]. The simpler way to overcome this barrier is to implement a hybrid model of delivery, where online sessions are mixed with in-clinic encounters for hands-on approaches. For manual therapy, clinicians can also consider teaching self-mobilization techniques to patients, which can contribute to the promotion of self-management and self-efficacy for patients [90]. Effective communication between patients and clinicians helps the therapeutic alliance and promotes patient involvement in collaboration with the therapist as integral to a patient-centered approach [56, 91]. The patient-centered care includes providing individualized care based on the context of the patient and their preferences, and shared decision-making [56, 92].

Telerehabilitation is a good option for self-management strategies, and it can promote patient empowerment, education, and independence [57, 93, 94]. What Lupton (2013) [95] describes as the “digitally engaged patient” (i.e., a patient that actively participates in health decision-making processes and self-care) is reinforced along the discourse of telerehabilitation. In the attempt of taking control over their health, lay people adopt behaviors of seeking health-related information online and use digital technologies to gather information (e.g., about physiology, lifestyle, etiology or condition-specific treatments) and participate in support groups [95]. However, health information online is often non-scientific, non-evidence-based, and sometimes biased by conflicts of interests (teaching of private courses, partnerships with companies) [96]. Some studies have investigated the content analysis of online information about low back pain [97], spine surgery [98], anterior cruciate ligament [99], and health jornal infographics [100], and observed that the content is commonly not aligned with the best available evidence. Most online information does not report sufficient information to allow readers to interpret the study findings [100]. The inaccurate information could mislead patients' treatment choices and create unrealistic expectations [99]. Patients and clinicians should be careful when searching for health online information. There is a clear need that representative healthcare entities recognize the importance and their role in providing evidence-based information for both, the professional and the general public [96, 97].

Besides the challenge of the quality of health-related information online, the use of the internet for this purpose seems to be restricted to a few social groups [101]. Women, white people, and individuals with a higher educational level are more likely to seek health-related information online [101]. Patients with lower health literacy apparently avoid seeking health information beyond the health encounter and show difficulties in playing the “engaged patient” role. Lower health literacy levels are associated with higher ages, lower educational levels, and lower incomes [102]. Furthermore, sociological branches discussing digital health technologies claim that patient empowerment is linked to greater self-responsibility and they assume the possibility of patients not wanting to be responsible for their health and preferring to leave health-related issues in the hands of their health professionals [95, 101, 103]. Beyond that, further research is needed to evaluate whether health-related information online may improve patients' experience and health outcomes [96].

Given the technological nature involved with telerehabilitation, low digital health literacy levels may also limit patient participation. The concept of digital health literacy is related to the capability of usage and usage outcomes, going beyond the sole access to the internet [104]. Patients with low digital health literacy may report unfamiliarity with the digital environment, difficulties in navigating, and the need to rely on family and friends to enable participation in telerehabilitation initiatives, elements that may further lead to patient disengagement, feeling of frustration and dependence on others, or poor patient participation.

Difficulties with the digital environment may also be present at the health professionals’ end in some cases, the adoption of telerehabilitation initiatives requires a new modus-operandi from clinicians in terms of workflow and demands (e.g., knowledge of information technology, secure platforms, data privacy) [101, 105]. Resistance may appear towards performing the initial assessment remotely, mainly due to the lack of touch and impossibility of assessing the patient using hands-on maneuvers [105, 106]. Barriers to telerehabilitation also encompass environmental and resource domains: i) physical space issues, such as inadequate equipment (i.e., mat, weights) and poor atmosphere to perform proposed activities (i.e., enough light, silence) [65, 94]; and ii) technological issues, such as internet malfunctioning or unstable, low quality of video or audio, and audio that is out of sync with the video [57, 65, 106]. Those barriers can be present both at the patient- and health professionals- end.

Moving from the individual to the interactional sphere, telerehabilitation may pose additional challenges to the health professional-patient relationship [65]. Communication becomes the first and foremost aspect of the health encounter due to the impossibility to touch and the limited presence of visual cues [107]. Literature highlights the use of probes, probing, and communication techniques such as teach-back [107, 108]. The aim is to deepen into the acquisition of information, further explore what is brought up by the patients, and check if the information was correctly understood. A second challenge comprises the construction of therapeutic alliance and trust in the digital environment, core elements of treatment adherence and participation [109]. Trust relationships are cultivated by interaction, the feeling of being listened to and reassured [109]. Therefore, health professionals need to communicate beyond the transmission of health-related information (e.g., symptoms, diagnostic, and prognostic) and active listen, provide feedback, and adopt an empathetic position regarding patients’ experiences [107, 109].

Further barriers to telerehabilitation strain the domains of infrastructure (i.e., the need for broad internet coverage, especially in locations with geographical distances) and public policies (i.e., guarantee access to an internet connection that is stable, of high quality, and available for those in need; reimbursement of telerehabilitation services by private health insurances; and practicality of offering telerehabilitation in public health services). Despite those barriers being outside the health professional-patient realm, they affect who can access telerehabilitation and the quality of the service delivered. Telerehabilitation is rapidly advancing along with the discourse of being an alternative to reduce healthcare costs [95, 110]. However, the democratization of access and the maintenance of healthcare quality remain the goals. Challenges and barriers must be carefully accounted for to prevent healthcare underinvestment and precariousness and contribute to a health service that is inclusive and relevant from health professionals' and end users’ point of view.

## Curriculum and education for the future

Despite the recent interest in telerehabilitation and telehealth, strategies to provide more guidance to clinicians, such as teaching and curriculum in the area have not been comprehensively studied yet. It is important to enabling future health professionals to achieve technical knowledge through digital literacy, technological learning, skills development, ability to care for the patient, clinical knowledge, and practice through the virtual environment [111–113]. A large academic health system in New York City showed a 683% increase in video visits within the first month period of the COVID-19 pandemic [114]. The Association of the American Medical Colleges published data from 2019–2020, in which 60% of the schools participating in the survey included telemedicine as a mandatory or elective subject [115]. They also conducted a Delphi study, reaching a consensus on the experiences and skills examined as prerequisites for today's clinicians who need to provide good care and meet an expanding virtual care demand [116].

Curricula for telehealth in medicine for undergraduate education were highlighted in a review published recently, showing that there are several ways of teaching telemedicine, through synchronous, asynchronous, face-to-face classes and meetings with real or simulated patients via virtual means [117–121]. Some of the studies in this systematic review show the purpose of the curriculum is to expose the student to equipment and materials, applications, and specific technologies of telehealth. As well as carrying out practices of simulated meetings through video conference, performing an adequate consultation with the patient, analyzing the reduction of costs by using the telemedicine service, and understanding the capabilities and limitations of telehealth in providing services to patients [117, 119, 120]. The training approach in a specific platform for virtual assistance was reported by another study, comprising online assistance, videos of simulated cases for prior training of the student, a virtual calendar for scheduling appointments, and ending the training with an assessment of the general performance of the students [121]. Important content such as history, applications, legislation and ethics in telehealth, safety, usage etiquette, camera angles, correct adjustment of the environment (e.g., lighting and room design), noise adequacy, as well as doctor-patient relationships, selection of patients and their perceptions, are essential items in a teaching telehealth program [120]. Most of the curricula proposed were applied from the second to the fourth year of the undergraduate medicine course, being useful in developing skills and knowledge acquired in telehealth [118, 122]. Table 3 presents the main competencies and skills necessary for a telehealth curriculum in higher education based on previous studies.

**Table 3:** The competencies and skills framework necessary to integrate the telehealth education curriculum

**Domain I—Principles of telehealth**
Definition and terminology
History of telehealth
Differences and similarities between virtual and face-to-face assistance, including risks and benefits
Objectives of the telehealth offer
Patient indications and screenings
Evidence-based practice in telehealth
Description of the fiscal impact of telehealth care for the health system, the provider and the patient
**Domain II—Care planning and management**
Obtaining patient information, for example the address, if you need an emergency service
Patient instructions related to the appointment, such as office hours, technical details (lighting, camera position), professional contact information and platform settings used
Stimulation of patient self-management and self-care
Development of a patient care plan, checking whether the approach should be mixed, face-to-face care combined with telehealth
**Domain III—Assessment, diagnosis and treatment**
Qualification of the student to perform assessment, clinical examination and diagnosis of the patient in the virtual environment
Preparation of a treatment plan and delivery of treatment via telehealth
Applicability of services via synchronous or written materials and/or asynchronous videos
Practical classes with the development of simulated and real care, promoting the clinical experience of virtual care
**Domain IV—Adequacy of the Environment**
Usage etiquette instructions, such as: tone of voice, eye contact, body language, greetings and closings, attention to interruptions, professional attire
Instruction of the service location, such as: clean space, no distractions
Setting the physical environment for lighting so that the patient visualizes the therapist
Camera use instructions: angles and framing
Influence of noise in the therapist's and patient's environment
**Domain V—Professionalism**
Empathetic communication, creating a therapist-patient relationship, bonding with the patient, caregivers and family members
Creating safe care through listening and trusting with the patient
Align expectations and goals of care between therapist and patient
**Domain VI—Legal Aspects**
Understanding Federal and Local Laws in Providing Telehealth Service
Understanding Federal and Local Laws Regarding Telehealth Service Reimbursement
Patients' rights in accepting or refusing virtual care
**Domain VII – Patient Privacy**
Generate patient privacy during virtual care
Need to obtain patient consent if there are videos or photos of the service
Confidentiality of information registered in the virtual service and how to store it
**Domain VIII—Patient Safety**
Patient safety risks when receiving care via telehealth, identifying the patient's health conditions and the physical environment
Identification of the risks, benefits and limitations of the patient when receiving care via telehealth
Patient instructions for setting up the physical environment for care
Recording in medical records of telehealth care
Understanding data security requirements in the use of telehealth platforms, storage of patient data in compliance with federal, state, and professional agencies
Early identification of emergency needs and referral of the patient to the emergency room
Identification of patient companions, if you need help in the assessment or care, and ensure safety when necessary
**Domain IX—Access and Equity**
Equity in patient care, assessing socioeconomic gaps in access to virtual care
Considerations regarding cultural, social and digital literacy barriers in virtual service
**Domain X—Technology**
Operation on existing software to provide a virtual service
Hardware operation enabling telehealth delivery
Preparation for use and technical troubleshooting
Security of the virtual environment and service platforms

These findings are similar to a previous review of telehealth education and training, which addressed topics including curriculum inclusion such as terminology, clinical applications, evidence bases and technology, using conventional methods and online education [111]. However, the conclusions are limited, especially due to the low number of studies in this area. Recently, the core capabilities that healthcare professionals need to provide quality video care were researched in a modified e-Delphi study [123]. This study showed that physical therapists need seven domains to provide online care: compliance; patient privacy and confidentiality; patient safety; technology skills; telehealth delivery; assessment and diagnosis; and care planning and management [123].

Thus, the need for a structured curriculum supported by scientific data is evident, in order to improve education and guidance to health professionals in providing telehealth and telerehabilitation interventions.

## Conclusion

Telerehabilitation plays an important role in improving health outcomes for musculoskeletal disorders and can promote patient engagement in health care, offering new opportunities to educate patients and facilitate the process of a behavior change in a healthy lifestyle. Telerehabilitation has been widely used in the world, however, to ensure a better practice of telerehabilitation, the clinician should ensure a better adaptation to the process of implementing telehealth in clinical practice and certify safety and access, environment, and communication, appropriately assessment and therapeutic prescription.

The main needs in telerehabilitation are the inclusion of it in health curriculums in higher education, standardization of telehealth-related terminologies, and the development of cost-effectiveness and implementation trials, especially in low- and middle-income countries where access, investments and digital health literacy are restricted.

## Data Availability

Not applicable.

## Abbreviations

COVID-19: Coronavirus disease
HIPAA: The Health Insurance Portability and Accountability Act
